# Challenges and novel approaches for investigating molecular mediation

**DOI:** 10.1093/hmg/ddw197

**Published:** 2016-07-20

**Authors:** R.C. Richmond, G. Hemani, K. Tilling, G. Davey Smith, C.L. Relton

**Affiliations:** ^1^MRC Integrative Epidemiology Unit, University of Bristol, UK; ^2^School of Social and Community Medicine, University of Bristol, UK

## Abstract

Understanding mediation is useful for identifying intermediates lying between an exposure and an outcome which, when intervened upon, will block (some or all of) the causal pathway between the exposure and outcome. Mediation approaches used in conventional epidemiology have been adapted to understanding the role of molecular intermediates in situations of high-dimensional omics data with varying degrees of success. In particular, the limitations of observational epidemiological study including confounding, reverse causation and measurement error can afflict conventional mediation approaches and may lead to incorrect conclusions regarding causal effects. Solutions to analysing mediation which overcome these problems include the use of instrumental variable methods such as Mendelian randomization, which may be applied to evaluate causality in increasingly complex networks of omics data.

## Introduction

New technologies permit the genotyping and profiling of gene expression, epigenetics and metabolites, allowing the collection of high-dimensional molecular phenotype data on a large number of individuals. This “omics revolution” (1,2) offers the potential to vastly improve the granularity of measurements related to the processes of normal development and disease pathogenesis.

**Figure 1.**
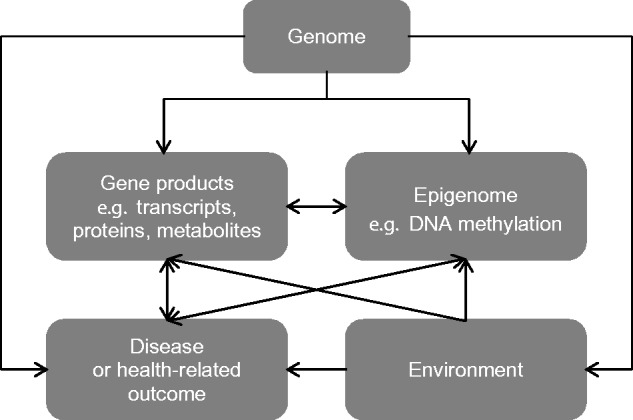
The interplay between genomics, other **“**omics**”** and environmental factors in relation to disease or health-related outcomes.

Recent applications of omics technologies within large-scale population-based studies present new opportunities for identifying novel biomarkers for both risk factors and disease. Furthermore, different forms of omic data can be combined with increasingly complex models (3) and may help to interrogate otherwise opaque networks in confirming observed risk factor and disease associations from observational epidemiology and identifying new ones (4) (Fig. 1).

However, as these molecular intermediates are influenced by both endogenous and exogenous factors and by disease processes, they are prone to the many limitations of observational epidemiological study including confounding, bias and reverse causation (Box 1) (5). We are therefore presented with the challenge of understanding the causal nature of correlations between measures of interest. Statistical methods are required to dissect causal relationships and to construct a causal framework involving molecular intermediates (6,7).

## What Is Mediation Analysis and Why Is Understanding Mediation Useful?

**Figure 2.**
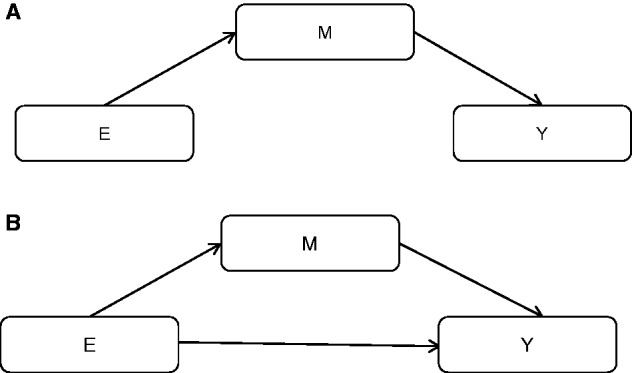
A simplistic representation of mediation. (A) Complete mediation - M is the only mechanism by which E can change Y. (B) Partial mediation - In practice, it is more likely that E has an effect on Y other than those operating by changing M. Mediation aims to partition the total (causal) effect of E on Y into mediated effects (effects that operate by changing the mediator, M) and non-mediated effects.

A mediator (M) is a variable that is on the causal path from an exposure (E) to an outcome variable (Y). It causes the outcome and is itself caused by the exposure. There are a variety of statistical methods that have been introduced for analysing mediation, from simple regression-based systems and structural equation models to more novel parametric and semi-parametric methods (8), and these have been widely implemented (Fig. 2).

Understanding mediation is useful for identifying potential modifiable risk factors lying between an exposure and an outcome which, when intervened upon, will block (some or all of) the causal pathway between the exposure and outcome. For example, elevated levels of non-fasting remnant and LDL cholesterol levels are modifiable intermediates of cardiovascular disease. These may be intervened upon to alter the downstream risk of cardiovascular disease, when underlying risk factors are either difficult, as in the case of adiposity (9), or indeed impossible to alter, as in the cases of the underlying genetic factors related to cholesterol levels (10).

Mediation approaches have been adapted to understanding the role of molecular intermediates in causal pathways, using high-dimensional omics data (4,11–18). However, these approaches have been applied with varying degrees of success as each approach has different strengths and challenges due to their underlying assumptions.

## Exposure – Outcome Mediation

**Figure 3.**
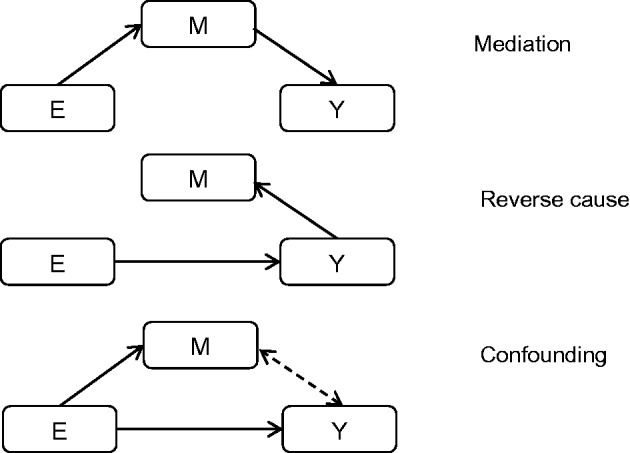
Distinguishing mediation from reverse causation and confounding In a situation of mediation, the effect of the exposure (E) on an outcome (Y) is mediated through an intermediate (M). In a situation of reverse cause, E influences Y which then has an effect on M. In a situation of common cause (confounding), E has an independent effect on both M and Y, so inducing a spurious association between M and Y.

One of the most widely cited approaches for evaluating mediation in an epidemiological setting is that originally outlined by Baron and Kenny (19). This regression-based approach may be applied to distinguish a mediated effect of the exposure (E) on an outcome (Y) through an intermediate (M) from both a consequential (reverse cause) effect and a common cause (confounding) effect (Fig. 3), through the application of four tests:

1. E is associated with Y
2. E is associated with M
3. M is associated with Y after adjusting for E
4. E is independent of Y after adjusting for M

The Sobel test may then be used to indicate whether the decrease in the effect of E on Y after adjusting for M is “statistically significant”. If this test provides evidence for mediation, the proportion of the effect of E on Y that is mediated by M can be calculated.

While this approach is widely implemented, it is known to be problematic because it is highly dependent on a number of strong assumptions, the measurement characteristics of the variables and on reliable identification of causal effects. Some such often overlooked assumptions are that (i) both Y and M are continuous; (ii) there are no unmeasured confounders of E and Y or of M and Y; (iii) E must not cause a confounder of the M-Y association; (iv) the correct functional form has been specified for each model (e.g. linearity); (v) there are no interactions between E and M on Y; and (vi) there is no measurement error (20). Here, measurement error is the difference between a measured value of E, M or Y and its true value, which could be due to either imperfect measurement (e.g. measuring weight using a standard set of scales) or fluctuating about an underlying “true” value (e.g. day-to-day variation in weight about the individual’s underlying average weight), or both. Furthermore, this method can only be used under the assumption of complete mediation as in a situation of partial mediation, the fourth condition will not hold.

Further methods have been developed to allow much more flexible modelling than the traditional Baron and Kenny approach and allow for a more general outcomes framework, distribution-free estimates of mediated effects, interactions and intermediate confounding (20,21). Such methods include linear equations, structural equation models, marginal structural models and G-computation. However, while these approaches offer more flexibility (e.g. allowing non-continuous variables and interactions between E and M in their effect on Y), they also require strong assumptions, specifically related to no measurement error or unmeasured confounding. If the assumptions are not satisfied, these methods may also lead to incorrect inference (22–26).

Nonetheless, some of these approaches have been readily applied in the setting of the molecular mediation without much consideration being given to the underlying assumptions and thus may have led to spurious results and interpretations. For example, large epigenome-wide association studies (EWAS) have identified associations between smoking and DNA methylation (27), and lung cancer and DNA methylation (15). Interestingly, CpG sites in the *AHRR* region have shown the largest signals of differential methylation in both these EWASs. These findings have driven subsequent analyses to investigate whether environmentally modified DNA methylation play an important role in the aetiology of cancer, through the use of mediation analysis.

One recent study used a causal mediation technique of G-computation to assert a mediating role of lower *AHRR* methylation in the association between smoking and lung cancer (15). Strikingly, the mediation analysis applied in this study identified that 32% of the total effect was mediated by differential methylation in the *AHRR* region. However, the study analysts also found that 31% of the total effect was mediated by methylation in a CpG in *F2RL3*, another site implicated in both smoking and lung cancer EWAS. Together, these two sites in *AHRR* and *F2RL3* explained a total of 37% of the total effect, which is lower than the proportion anticipated, given that these two genes are independent and act through different biological pathways.

One potential explanation for these findings is that the association between methylation and lung cancer might just reflect the known causal effect of smoking on lung cancer, as DNA methylation is a strong biomarker for smoking (28). Mediation analysis may lead to a spurious inference due to measurement error, in this instance in the exposure, whereby self-reported smoking is more error-prone than objectively-measured DNA methylation, leading to residual confounding of the intermediate – outcome association.

## Genetic Variant – Outcome Mediation

A widely-used approach for establishing causal relationships with molecular intermediates is the causal inference test (CIT) (29). This test builds on the ‘Causality Equivalent Theorem’ (30) to infer causal indirect effects of a genetic variant on an outcome. It is analogous to the Baron and Kenny approach in its reliance on a series of models to statistically test conditional independencies between covariates in order to distinguish a mediated effect of the genetic variant (G) on an outcome (Y) through an intermediate (M) from a reverse cause and a common cause (pleiotropic) effect (7,29,31). Therefore, replacing E (exposure) in the Baron and Kenny approach with G (genetic variant), the required tests are:

1. G is associated with Y without adjusting for M
2. G is associated with M after adjusting for Y
3. M is associated with Y after adjusting for G
4. G is independent of Y after adjusting for M

This approach has typically been applied to understand the extent to which molecular processes mediate the effect of quantitative trait loci (QTLs) on the risk of a particular disease. By focusing on the assessment of the genetic component of the molecular intermediate, this avoids limitations in the observational epidemiology setting of potential confounding from environmental factors on the intermediate – outcome relationship and also the influence of reverse causation, whereby changes in the outcome may influence the intermediate factor. In addition, some other qualities of genetic variants which make them useful in the causal inference analysis are that they are not influenced by reporting bias and are subject to relatively little measurement error.

By using a genetic variant as a causal “anchor” to dissect causal relationships, the causal inference test has close links with Mendelian randomization (MR) (5,32), a method which will be discussed in more detail later in this review, although with the use of a different modelling approach. Given its ease of application, the causal inference test has been used to evaluate molecular mediation in a range of omics settings (6,11,16,33). However, this test is limited by its emphasis on an “omnibus statistical test” (29) which is reliant on a p-value for asserting a causal effect, rather than providing an estimate for the magnitude and precision of the true causal effect (34).

Furthermore, this approach is also vulnerable to measurement error, which can be in either the mediator or the outcome, in those steps 2) to 4) of the causal inference test (see above) which involve adjustment in the regression models.

In particular, the presence of measurement error can make it hard to delineate a situation of true mediation from that of horizontal pleiotropy (defined in Box 2), whereby the genetic variant influences the outcome through pathways other than those containing the mediator. The problem of incorrectly identifying pleiotropy can be illustrated with an example, whereby a genetic variant associated with cholesterol levels is strongly associated with coronary heart disease even after adjusting for measured cholesterol (35). While the causal inference test would infer from this that cholesterol is not fully mediating the effect of this genetic variant on risk of coronary heart disease, in reality this situation probably arises because the genetic variant represents a life-long elevated risk of cholesterol levels compared with a single, poor measure of cholesterol acting as the mediator in this situation (Table 1).

**Table 1.** Causal inference test analysing the causal effect of total cholesterol on coronary heart disease (CHD) using the rs72658867 LDLR splice variant SNP as a causal anchor

Condition	P-value
CHD associated with LDLR	4.33x10^−3^
CHD associated with total cholesterol given LDLR	<1.00x10^−5^
Total cholesterol associated with LDLR given CHD	<1.00x10^−5^
LDLR **independent** of CHD given total cholesterol	>0.99
Total cholesterol causes CHD (omnibus P-value)	>0.99

Analysis was performed using cholesterol and CHD data from the Copenhagen General Population Study using 95,275 individuals. Data described here http://www.nature.com/nature/journal/v526/n7571/full/nature14962.html.(36) Analysis performed using the R/cit package and the *cit.bp* function. *P*-values represent the strength of evidence for each condition of the causal inference test. In this situation, three of the four conditions of the causal inference test are satisfied although the fourth is not (*P* >0.99), as *LDLR* is associated with CHD even after adjusting for total cholesterol. The omnibus test therefore selects this largest *P*-value, which in this case is used to reject the hypothesis that total cholesterol causes CHD.

Measurement error can also lead to erroneous results from the causal inference test due to reverse cause, whereby the genetic variants influence the proposed mediated factor indirectly through an effect of the outcome (Fig. 3). This may occur in a situation where a genetic variant is found to be associated with a proposed mediator but is of unknown biology. Here it is possible that the genetic variant is directly related to the outcome and only indirectly to the proposed mediator through the causal effect of the outcome on the intermediate factor. For example, greater adiposity is known to have a causal effect on levels of the inflammatory biomarker, C reactive protein, but not vice versa (37,38). With adequate sample size and measurement precision, any genetic variant related to body mass index will be related to CRP because of this causal effect (37,38). Furthermore, in the absence of knowledge about the functionality of the genetic variants, true adiposity variants may be assumed to be directly related to CRP levels, which may lead to incorrect causal inference.

## Use of Mendelian Randomization for Mediation Analysis

**Figure 4.**
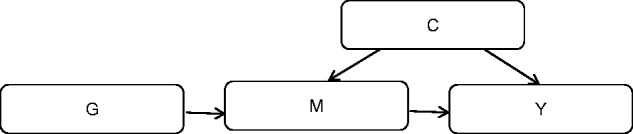
Schematic representation of Mendelian randomization to assess the causal effect of a molecular intermediate Mendelian randomization can be used to test the hypothesis that molecular intermediate M has a causal effect on outcome Y, given that the genetic variant G is associated with the intermediate phenotype of interest, has no association with the outcome except through the intermediate phenotype and is not related to measured or unmeasured confounding factors (C).

Solutions to analysing mediation which overcome unmeasured or residual confounding, reverse causation and measurement error include the use of instrumental variable methods (8), of which Mendelian randomization (MR) is a form. In MR, genetic variants robustly associated with modifiable exposures are used to infer causality (5,32,39) by serving as instrumental variables which are not associated with various confounders of the exposure-outcome association and are not directly influenced by the outcome of interest (40,41) (Fig. 4).

The assumptions and application of Mendelian randomization analysis were outlined in detail in a recent review (5). In addition, this review outlined how the MR approach may be adapted to the setting of appraising causality of molecular phenotypes. However, with specific reference to gene-outcome mediation analysis, using the MR approach has two potential advantages over the CIT: 1) it allows for a formal test of the direction and magnitude of causality, rather than a p-value driven assessment, and 2) by using the genetic variant as an instrumental variable for the mediator, the correct direction of causality can be inferred even in the presence of measurement error in the mediator, as genetic variants are typically measured with high accuracy and will typically proxy for lifetime differences in exposures (39).

Limitations of MR have been discussed in detail in a recent review (5), which also highlighted some methodological developments to overcome these limitations. Specifically with respect to using MR to assess mediation, the main limitations of this approach are low power, potential pleiotropy of the genetic instruments and reverse cause.

While genetic instruments for molecular phenotypes often explain a large proportion of variance in these traits (Box 1), MR studies involving such intermediates are often of low power because of the availability of biological samples and the relative expense of measuring these phenotypes in large enough numbers. One recent means of enhancing power in Mendelian randomization analysis is with the use of a two-sample approach (50,51). This approach is particularly relevant for establishing the causal effect of a molecular intermediate, which only needs to be measured in a subset of individuals with genetic data, and then integrating these gene-exposure estimates with gene-outcome estimates obtained from larger studies to harness power. With respect to the latter, such estimates may be obtained from publicly available summary measures which are increasingly available for many large genome-wide association studies (51).

**Figure 5.**
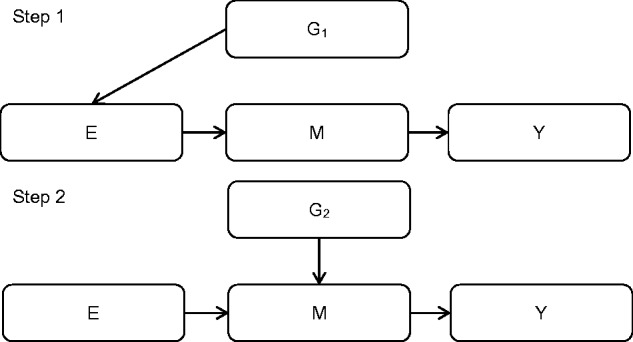
Schematic diagram of two-step Mendelian randomization In Step 1, a genetic variant, G_1_, is used to proxy for the environmentally-modifiable exposure of interest, E, to examine how this exposure influences in the intermediate, M, e.g. DNA methylation. In Step 2, a different genetic variant unrelated to the exposure, G_2_ is used to proxy for this specific difference in the intermediate, M, and relate this to the outcome of interest, Y.

In situations such as that outlined with respect to evaluating the role of a molecular intermediate (e.g. DNA methylation) in a known exposure-outcome relationship (e.g. smoking-lung cancer), it may be possible to obtain causal estimates from MR studies for all steps in the chain, e.g. from smoking to DNA methylation, and from DNA methylation to lung cancer, in a two-step Mendelian randomization approach (Fig. 5). Here the logic of MR can be extended to interrogate causality of a mediating effect using one genetic instrument to estimate the causal effect of the exposure on DNA methylation, and a separate independent instrument to estimate the causal effect of DNA methylation on the outcome. As variation in DNA methylation is associated with widespread local (*cis*) genetic variation, this provides the opportunity to use genetic proxies to probe causality between DNA methylation and particular outcomes using MR (5,42–44).

While the two-step MR method was initially posed for the delineation of mediation by specific epigenetic processes between environmental exposures and disease, it may equally be applied to a full range of potential mediators, such as transcriptomics, proteomics and metabolomics (45). In addition, while evidence of association in both steps of the two-step MR framework implies some degree of mediation, in its original form this method did not give a quantitative contribution of the mediator to the causal link explicitly. Extensions of network Mendelian randomization (10,45) allow for the magnitude of the direct and indirect effects to be estimated and can be used to obtain support for a number of testable hypotheses and degrees of association between increasingly complex networks. Such methods will be particularly useful for integrating omics data and challenging the “central dogma” of biological causation (46–49).

**Figure 6.**
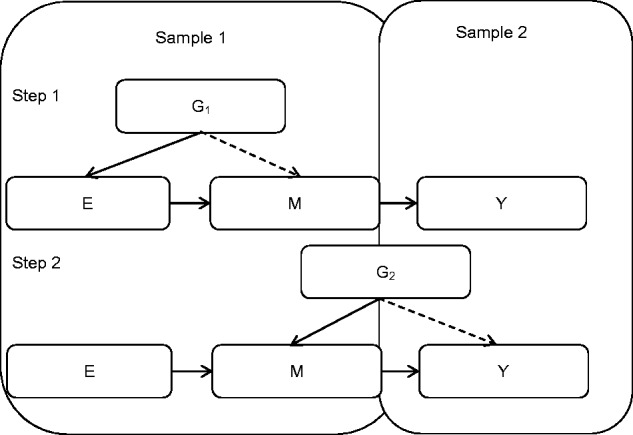
Schematic diagram of two-step, two-sample Mendelian randomization In the smaller Sample 1, the association of the exposure to the intermediate is established using an MR approach (using the exposure-related G_1_); and the association of an additional variant (G_2_, not related to the exposure) with the same intermediate is established. G_1_ and G_2_ should be identified in an independent study. In the larger Sample 2, the intermediate is shown to influence the outcome through the use of G_2_, which is related to the outcome. N.B. the dotted arrows represent the fact that these genetic variants, G_1_ and G_2_, influence the intermediate or outcome indirectly through the exposure or intermediate, rather than having a pleiotropic effect. In theory, G_1_ would also be found to influence the outcome indirectly through both the exposure and intermediate.

In addition, with regards to asserting mediation in an exposure-outcome setting, the two-step MR approach could be combined with the two-sample approach to powerfully and efficiently examine the extent of mediation in causal networks (5). First, the causal associations of both the exposure on the intermediate and of an independent variant on the intermediate could be established, and then in a larger population-based sample, the genetic associations with the disease outcome delineated (Fig. 6). This gives two-step MR an advantage over traditional mediation approaches which require the exposure, mediator and outcome to be measured in the same subset of individuals.

As with the causal inference test, complexity of associations between omic level intermediates and inadequate biological knowledge of the genetic variants associated with them pose a challenge to Mendelian randomization. Arguably, the biggest challenge to overcome is that of potential pleiotropy of the genetic instrument (Box 2). Approaches which have recently been developed to allow causal effect estimates in the presence of pleiotropy, and which are also particularly relevant to causal inference for molecular mediation, are described in more detail in Box 2.

In situations of reverse causation whereby a genetic variant may be causing the outcome which in turn causes the molecular phenotype, rather than vice versa, bidirectional Mendelian randomization using well characterized genetic variants for both the molecular intermediate and the outcome may be used to distinguish between these causal models (37,38). Alternatively, the use of the Steiger test may be able to provide evidence for the prevailing causal direction, based on the estimated variance explained by the SNPs in the molecular phenotype and the outcome, as long as measurement error in the molecular phenotype is lower than the product of the measurement error in the outcome and the causal correlation between the molecular phenotype and the outcome (66).

## Conclusions

Mediation analysis and causation are linked concepts and the former cannot be successfully applied without some consideration given to the latter (67). Care must be taken when conducting mediation analysis in making sure that the assumptions made in the causal model are justified (68). In particular, the assumptions of no unobserved confounding and no measurement error are often made in both conventional epidemiology (exemplified in the Baron and Kenny approach) and computational systems biology (69) (exemplified in the Causal Inference Test) which vitiate many of the models attempting to utilize measured phenotypes and which therefore can lead to erroneous inferences or conclusions being drawn.

Mendelian randomization approaches hold promise for investigating mediation without relying on such strict assumptions (10,43,45). Furthermore, such techniques focus on minimizing reliance on correlation statistics and maximizing quantitative causal interpretation by using genetic variants as causal anchors in situations of mediation. These approaches are increasingly being used in an automated, hypothesis-free fashion (51) and may be used to integrate multiple tiers of omics data in a causal framework. This offers potential for identifying novel risk factors and modifiable targets for intervention.

While MR is a helpful solution in some circumstances when considering molecular mediation, it is not a global solution and its application can be restricted due to the availability of genetic instruments. In addition, while MR makes less strict assumptions about confounding and measurement error, it does make other assumptions (39,70,71) which should be explored through sensitivity analysis (62,63). We would also advocate an integration of causal inference approaches and the triangulation of findings in the domain of high-dimensional molecular data to improve the identification of causal mediating effects (6).

Box 1. Identifying genetic proxies for ‘omics measures

Developments in genomics have driven the identification of many genetic variants associated with a wide range of exposures which have potential utility in MR analysis (5,52). As molecular intermediates are more proximal to genotype than downstream phenotypes (53) this boosts the statistical power to detect associations compared to more complex traits, as exemplified in genetic association studies.(54–56) Furthermore, studies have identified that many genetic effects on intermediates are highly stable across the life course (55) and between tissues (56). Such stability is useful when these genetic variants (or quantitative trait loci (QTLs)) are being applied as causal anchors. Catalogues of these QTLs are being made freely available.(55,56)

Box 2. Consequences of pleiotropy and potential solutions for Mendelian randomization analysis

Pleiotropy is the phenomenon by which a genetic variant may affect more than one phenotypic characteristic (57–59). There are two main mechanisms by which pleiotropy occurs: 1) a single locus influences a cascade of events e.g. a variant influences a particular molecular intermediate which causes perturbation in another phenotype (vertical pleiotropy) 2) a single locus directly influences multiple phenotypes e.g. via more than one post-transcriptional process (horizontal pleiotropy). Vertical pleiotropy has also been referred to as ‘mediated pleiotropy’ (60) and is the very essence of the Mendelian randomization approach, in which the downstream effects of a phenotype are estimated through the use of genetic variants that relate to that phenotype. On the other hand, horizontal pleiotropy is more problematic as it violates the assumption that the genetic instrument has no association with the outcome except through the intermediate phenotype being investigated and its presence can bias Mendelian randomization effect estimates.

One potential means of investigating potential pleiotropy is with the use of multiple genetic instruments. With an amassing number of independent instruments, it would be increasingly improbable that they would result in the same conclusion regarding a causal effect if they were all pleiotropic variants. In particular, the finding that all genetic variants have an effect on the outcome to the extent expected given their effect on the exposure can be used to support an assertion of no horizontal pleiotropy (5,61). If some variants deviate from this proportional effect, then the extent of directional pleiotropy can be investigated with the use of an approach known as “MR Egger” (62), and further derivatives including a weighted median approach (63), which can also be used to provide valid causal estimates even in the presence of pleiotropy.

A further approach used to separate independent effects of risk factors when multiple phenotypes are correlated with a particular genetic variant or set of variants is with multi-variable Mendelian randomization analysis (64) which provides a more promising alternative to analyses which attempt to isolate the effects of correlated phenotypes using regression-based approaches (65).
